# Lithium battery fault diagnosis by integrating improved EMD decomposition algorithm and 2DCNN

**DOI:** 10.1371/journal.pone.0344847

**Published:** 2026-03-17

**Authors:** Xiaofei Yin, Hui Wang, Xiangfei Meng, Haofei Zhu, Like Zhong, Wei Zhan, Yang Pu

**Affiliations:** 1 Energy Science and Technology Research Institute, State Power Investment Corporation, Shanghai, China; 2 Qinghai Photovoltaic Industry Innovation Center Co., Ltd., State Power Investment Corporation, Xining, China; 3 Sichuan New Energy Research Center, Chengdu, China; 4 State Key Laboratory of Coal Combustion and Low Carbon Utilization, Wuhan, China; Sunway University, MALAYSIA

## Abstract

With the development of science and technology, lithium batteries, as important energy storage devices, have become a research hotspot for fault diagnosis. In response to the shortcomings of traditional fault diagnosis techniques in processing complex signals and extracting key features, a high-performance lithium battery fault diagnosis model is constructed by combining the high-dimensional representation ability of a two-dimensional convolutional neural network (2DCNN) with the decomposition stability of an optimized empirical mode decomposition (EMD) method. The study first uses an improved EMD algorithm to process the voltage data of lithium batteries and extract fault features. Moreover, the processed data are input into 2DCNN model for training and classification. The experiment results showed that the fault feature information consistency of the research designed model was 98.7% when the iteration number reached 600. The feature recognition accuracy dropped to 99.2% when the image contained 70 features. The running time in all 7 data groups was significantly lower than other methods, with the highest being below 6ms. The results indicate that the designed lithium battery fault diagnosis model has improved the accuracy and efficiency of fault diagnosis. This can provide new technological means for lithium battery fault diagnosis and helping to promote the further development and application of lithium battery technology.

## 1. Introduction

Lithium-ion batteries (LIBs) have become the cornerstone of modern energy storage systems, powering everything from electric vehicles (EVs) to grid-scale storage due to their high energy density and efficiency [1]. The safe and reliable operation of these batteries is paramount, as failures can lead to severe safety hazards and economic losses [2,3]. This underscores the critical need for accurate and early fault diagnosis (FD) techniques. However, a significant scientific challenge persists in the field of lithium battery fault diagnosis (LBFD). Traditional FD methods often rely on handcrafted feature extraction from one-dimensional (1D) voltage or current signals [4]. These signals are typically non-stationary and non-linear, especially during early fault stages, making it difficult for conventional techniques to isolate subtle yet critical fault signatures from overwhelming noise and operational variations. Consequently, existing approaches frequently suffer from insufficient feature discriminability, low diagnostic accuracy, and poor generalization capability under complex operating conditions.

The primary objective of this study is to bridge this gap by developing a novel FD framework that effectively addresses the limitations of handling complex, non-stationary signals. To this end, it proposes a synergistic integration of an improved empirical mode decomposition (EMD) algorithm and a two-dimensional convolutional neural network (2DCNN). This combination is strategically chosen to leverage their complementary strengths: EMD is a powerful adaptive signal processing technique adept at decomposing complex signals into intrinsic mode functions (IMFs), thereby simplifying the subsequent feature analysis [5]. Meanwhile, 2DCNN excels at automatically learning hierarchical and spatial features from image-like data, demonstrating superior performance in pattern recognition tasks [6]. The central hypothesis of this work is that by first using an improved EMD to preprocess and structure the raw 1D signal into a more informative 2D representation, it can fully unlock the pattern recognition potential of 2DCNNs for highly accurate and robust FD.

The main contributions of this research are: (1) To propose an improved EMD algorithm designed to enhance the stability and robustness of decomposing non-stationary battery voltage signals. (2) To design a novel data conversion strategy that effectively bridges signal processing and deep learning by transforming the decomposed components into a 2D format compatible with 2DCNN. (3) To construct and validate an end-to-end LBFD model that demonstrates superior performance in diagnostic accuracy, feature consistency, and computational efficiency compared to existing methods.

The remainder of this study is organized as follows. Section 2 reviews related work. Section 3 details the proposed methodology, including the improved EMD algorithm and the 2DCNN architecture. Section 4 presents experimental results and discussions. Finally, Section 5 concludes the study.

## 2. Related works

LIBs are widely used in EVs due to their high energy density. However, their frequent charging and discharging processes, as well as their delayed dynamic response, can easily lead to battery aging and failure [7]. In the research of FD methods, Deng et al. [8] developed an intelligent fault recognition technology to address the complexity of EV battery FD. Experiments showed that this method had significant advantages in processing nonlinear and high-dimensional data. Liao et al. [9] proposed an FD method based on wavelet packet decomposition and Manhattan mean distance, which effectively extracted fault characteristic frequency components through wavelet packet decomposition technology. In deep learning methods, Xin et al. [10] developed a diagnostic method based on deep separable convolutional neural networks that could effectively detect blade fouling in marine tidal turbines. Liu et al. [11] proposed a convolutional neural network based on enhanced symmetric dot patterns, which improved the accuracy of rolling bearing FD. In hybrid method research, Shen et al. [12] designed an energy management strategy combining EMD and fuzzy logic control. Tan et al. [13] proposed an integrated voltage equalizer based on voltage multiplier technology. Li et al. [14] developed a fast adaptive EMD method. Qi et al. [15] proposed an FD method combining EMD, deep learning, and knowledge graphs. Inturi et al. [16] developed a composite method based on discrete wavelet transform and EMD. Additionally, the research by Kikuta et al. [17] in image processing technology provided important references for FD. Although significant progress has been made in existing research, several shortcomings remain. Most methods are designed for specific faults and have limited generalization capability. There is insufficient adaptability and robustness in complex working conditions, and there is a lack of effective early fault warning capabilities. Additionally, there is inadequate consideration of battery-specific fault mechanisms.

Compared with existing research, most methods exhibit significant limitations when addressing complex and variable lithium battery fault scenarios. On the one hand, these approaches are primarily designed for specific fault types, lacking universality and struggling to adapt to diverse fault patterns in practical applications. On the other hand, traditional methods are not robust enough to handle complex operating conditions, such as strong noise and dynamic loads. They fail to detect subtle faults early on and do not adequately integrate battery-specific degradation mechanisms. Additionally, many methods still rely on manual feature design or complex parameter tuning, which limits their application efficiency in real-time diagnostic scenarios. In the field of battery fault diagnosis, various data-driven approaches have been explored. Among them, methods based on Manhattan distance [36]and voltage difference analysis [37] belong to classical feature engineering techniques. They use statistical measures such as mean absolute deviation and inter-cell voltage differences as health indicators. Although computationally simple and intuitive, these methods lack sensitivity to early-stage nonlinear faults embedded in complex noise, and their reliance on manually set thresholds and simplified signal models makes them prone to failure under dynamic operating conditions. More advanced frameworks, such as Multi-Scale Kernel Principal Component Analysis – Local Outlier Factor (Multi-Scale KPCA-LOF), attempt to address nonlinear and multi-resolution characteristics by projecting data into kernel space to capture nonlinear relationships, combined with multi-scale analysis and local density assessment for anomaly detection [38]. However, such approaches still face several challenges: they require substantial domain expertise for kernel function selection and scale definition, involve multi-stage optimization that complicates deployment, and struggle to learn highly discriminative spatiotemporal features in an end-to-end manner directly from raw or lightly processed signals. To address these issues, this study proposes an integrated deep FD architecture for LIBs. By introducing white noise assistance and an integrated averaging strategy, the research effectively mitigates modal aliasing problems in traditional EMD, thereby enhancing signal decomposition stability and reliability. In terms of data structure, it develops a mechanism to convert 1D feature modal components into 2D feature matrices, providing optimized input formats for advanced feature extraction. At the system level, the architecture establishes an end-to-end diagnostic workflow that combines enhanced signal processing with a 2DCNN for feature learning. This significantly improves diagnostic accuracy, enhances overall system robustness, and increases operational efficiency.

## 3. Methods and materials

**Fig 1:**
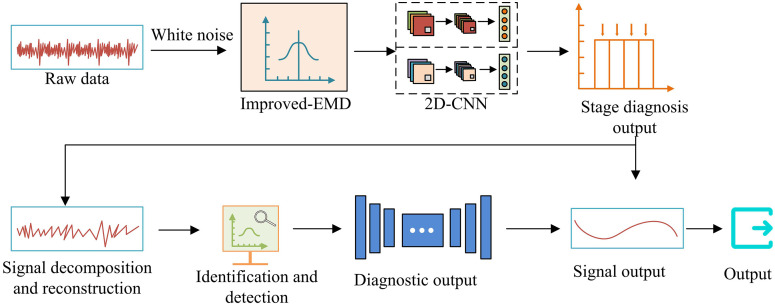
Systematic schematic diagram of the overall architecture.

This section provides a comprehensive framework and technical specifications for the proposed LIB FD model. As illustrated in Fig 1, the model features an end-to-end diagnostic process that integrates enhanced EMD with 2DCNN. The study focuses on detecting several critical and representative faults in LIB systems, including initial short circuits, capacity degradation, electrode material spalling, thermal runaway, and abnormal charge/discharge cycles. These faults are typically caused by manufacturing defects, long-term aging, and thermal abuse. Their early-stage characteristics often remain extremely subtle and obscured by noise within complex voltage signals. Therefore, the core challenge of this research is achieving precise, early detection of these faults, particularly initial micro-short circuits and performance deterioration. Subsequent sections will detail the key components of this diagnostic workflow. This section delineates the overall framework and specific technical details of the proposed LBFD model. The entire methodology follows a coherent pipeline, as illustrated in Fig 1, which provides a schematic overview of the integrated improved EMD and 2DCNN approach for LBFD.

As depicted in Fig 1, the methodology consists of four major stages: Data acquisition and preprocessing: Raw voltage signals are collected from LIBs under various operating and fault conditions. Signal decomposition and feature reconstruction: The raw signals are processed by the improved EMD algorithm to generate stabilized IMFs. The key IMFs containing the most discriminative fault information are selected and reconstructed into a 2D feature matrix. Deep feature learning and classification: The constructed 2D feature matrix is fed into the 2DCNN model, which automatically learns spatial-temporal features and performs fault classification. Diagnosis output: The model outputs the fault type and probability. The subsequent subsections will elaborate on the technical specifics of each core component within this integrated pipeline.

### 3.1. Construction of operational topology structure for LIB system

**Fig 2:**
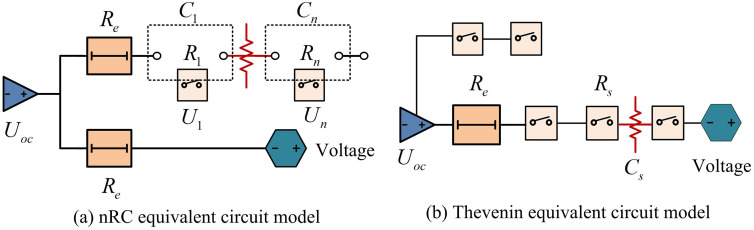
LIB model.

The ES mechanism of LIBs is based on electrochemical oxidation-reduction reactions, with the core being the reversible insertion and extraction of lithium ions between electrode materials. During charging, lithium ions are released from the positive electrode’s active material lattice and enter the negative electrode’s layered structure through the electrolyte medium. This results in a rich concentration of lithium ions in the negative electrode and a decrease in lithium ions in the positive electrode, thus creating a potential difference. To maintain charge balance, an equal number of electrons move through an external conductor to the negative electrode, forming a current cycle. During the discharge phase, the process undergoes a thermodynamic reversal. Lithium ions detach from the negative electrode and re-embed into the positive electrode’s lattice structure, releasing stored chemical energy and prompting electrons to flow through the external circuit [18,19]. The common LIB model is shown in Fig 2.

In Fig 2, $R_{e}$ represents equivalent internal resistance, $R_{s}$ represents polarization internal resistance, $U_{oc}$ represents voltage source, and $C_{s}$ represents polarization capacitance. Fig 2(a) shows the equivalent circuit model of nRC, and Fig 2 (b) shows the equivalent circuit model of Thevenin. The Thevenin equivalent circuit model can effectively simulate the charging and discharging process of LIBs [20]. The frequency domain transfer function of the equivalent circuit model is shown in equation (1).


$$U\left(s\right)=U_{oc}\left(s\right)-I\left(s\right)(R_{e}+\sum_{i=1}^{n}\frac{R_{i}}{1+R_{i}C_{i}s})$$ (1)

In equation (1), I(s) means the load current of the LIB. s represents Laplace variable. $C_{i}$ represents dynamic capacitance. U(s) represents the load voltage. $R_{i}$ represents dynamic resistance. $R_{i}$ stands for Laplacian variable. The mathematical model of LIBs is denoted in equation (2).


$$\left\{ \begin{array}{c} @l@x_{t}=f\left(x_{t-1},c_{t}\right)+\delta_{pt}\\ y_{t}=g\left(x_{t-1}\right)+\delta_{ot} \end{array}\right.$$ (2)

In equation (2), $x_{t}$ represents the state variable of the system. t represents the current time. $c_{t}$ represents the control input of the system. $\delta_{ot}$ represents the measurement error of the sensor during the observation process. $\delta_{pt}$ represents random interference during the system state update process. $g\left(x_{t-1}\right)$ represents the observed nonlinear function. $y_{t}$ represents the measurement value at time t. The state of charge (SOC) of a battery is a key indicator that describes the remaining charge during battery use, and its calculation formula is denoted in equation (3).


$$SOC=\frac{C_{\mu }}{C_{e}}$$ (3)

**Fig 3:**
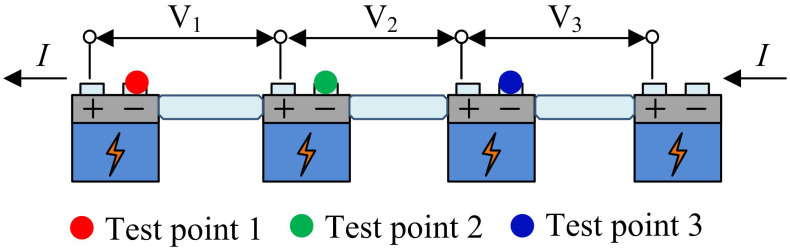
Simplified FD model for battery pack.

In equation (3), $C_{\mu }$ refers to the remaining available capacity of the battery for discharging at rated current. $C_{e}$ represents the rated capacity. The study simplifies the connection method of the battery pack and constructs a simplified FD model for the battery pack, as denoted in Fig 3.

In Fig 3, I represents the operating current of the battery pack, and $V_{\text{1}}$ represents the battery voltage. During the assembly process of battery modules, individual batteries are connected in series topology to form ES units, and reliable circuit conductivity is achieved through metal conductors and bolt locking technology. When the test point changes, there is no change in $V_{\text{2}}$ and $V_{\text{3}}$, only $V_{\text{1}}$ changes. By using the measured voltage values, the exact location of the fault can be determined.

### 3.2. LBFD based on improved emd decomposition algorithm

From the perspective of the failure mechanism of lithium batteries, micro short circuits are essentially the initial development stage of internal short circuits, and the two have the same fault occurrence mechanism. Usually, mechanical, electrical, and thermal abuses are the three main causes of internal short circuits in batteries [21]. Mechanical abuse of lithium batteries is usually caused by external forces or foreign objects penetrating the inside of the battery, resulting in deformation. The thermal abuse of lithium batteries may result in significant alterations to the battery separator, including excessive shrinkage or even complete collapse. This phenomenon can result in the positive and negative electrodes coming into direct contact with each other, thereby inducing internal short circuits within the battery. Electrical abuse can result in metal deposits forming inside the battery, which can cause internal short circuits. Internal short circuits may also be caused by impurities and particles mixed in during the manufacturing process of electrode materials or by burrs generated by improper cutting of electrode sheets [22,23]. To ensure the battery system operates safely and stably, a voltage data processing method grounded on EMD is proposed for LBFD. The EMD method can be utilized to decompose the initial signal and obtain the time-domain characteristics of the fault signal, as shown in equation (4).


$$x\left(t\right)=\sum_{i=1}^{n}E_{i}+W_{i}$$ (4)

In equation (4), $W_{i}$ represents the residual function, and $E_{i}$ represents the variance contribution rate. The standard deviation can determine whether the decomposition of the vibration source signal is complete, as expressed in equation (5).


$$S_{D}=\sum_{t=0}^{\tau }[\frac{\gamma_{1(k-1)}\left(t\right)-\gamma_{1k}\left(t\right)}{\gamma_{1(k-1)}\left(t\right)}{]}^{\text{2}}$$ (5)

In equation (5), $y_{1k}\left(t\right)$ represents the value of the first IMF obtained after the k th screening iteration at time point t. $\gamma_{1(k-1)}\left(t\right)-\gamma_{1k}\left(t\right)$ represents the difference between the results of two adjacent iterations. To address the limitations of traditional EMD, this study enhances the EMD algorithm by introducing white noise and averaging multiple decompositions. The amplitude of the white noise sequences is typically set as a fraction of the standard deviation of the original signal (e.g., 0.1–0.3 times) to ensure effective mode separation without overwhelming the signal. The frequency distribution is uniformly distributed across the entire frequency band to comprehensively assist in decomposition. The addition ratio is empirically determined through iterative trials that balance decomposition stability and computational cost. Common practice is to add 50–200 independent noise realizations and average their results [24]. Specifically, multiple distinct white noise sequences are incorporated into the original signal, followed by numerous decompositions. Compared with the standard EMD, the improved EMD algorithm proposed in this study can reduce the degree of modal aliasing and suppress the non-physical spectrum overlap between different eigenmodal functions through white noise assistance and integrated averaging strategy, thus achieving more distinct signal decomposition and providing more reliable time-frequency components for subsequent feature extraction. Specifically, multiple independent Gaussian white noise sequences are generated with amplitudes set proportionally to the standard deviation of the original signal. The coefficient typically ranges from 0.1 to 0.3. These noise sequences are successively added to the original signal to form a noisy signal ensemble. Each noisy signal is decomposed independently using EMD. The corresponding IMF components obtained from each decomposition are averaged by mode. This preserves the coherent structure of fault features while canceling out random noise. In this mechanism, white noise acts as uniform “dithering” during the sifting process to help separate closely spaced frequency components. The averaging operation then suppresses residual noise and stabilizes IMF boundaries. This approach effectively suppresses mode mixing, thereby enhancing the stability and robustness of the decomposition process. By doing so, the fault features extracted become more distinct and precise.

In this research, the improved EMD algorithm is employed to decompose lithium battery voltage data. The decomposition layers are dynamically determined based on the data characteristics, usually resulting in 3–5 IMF components and one residual term. During data reconstruction, it is crucial to retain the IMF components and residual terms that contain valid features to preserve the fault characteristic information. The white noise sequences are defined in equation (6).


$$x_{i\left(t\right)}=x_{\left(t\right)}+n_{i\left(t\right)}$$ (6)

In equation (6), $x_{\left(t\right)}$ represents the original signal and $n_{i\left(t\right)}$ denotes white noise. For each modal function component, the average of the modal function components under all corresponding noise sequences is calculated, as shown in equation (7).


$$c_{j\left(t\right)}=\frac{\text{1}}{N}\sum_{i=1}^{N}c_{ij\left(t\right)}$$ (7)

In equation (7), N represents the number of white noise sequences. $c_{ij\text{(}t\text{)}}$ represents the components of j functions obtained from the results. The improved EMD algorithm adaptively determines the decomposition layers based on the data characteristics, typically yielding 3–5 IMF components and one residual term. During data reconstruction, the IMF components and residual terms containing valid features are retained to preserve fault characteristic information. This method not only enhances the accuracy and reliability of fault feature extraction but also reduces dependence on the initial signal. As a result, it can better accommodate various noise environments and signal characteristics, providing a more robust data foundation for subsequent FD. To retain fault characteristic information, the IMF components and residual terms containing valid features should be retained during data reconstruction. 1D time series data is segmented into time windows and arranged into a two-dimensional matrix, forming a 2D data format suitable for 2DCNN input, which facilitates feature extraction.

### 3.3. Optimization design of LBFD model integrating improved EMD AND 2DCNN

**Fig 4:**
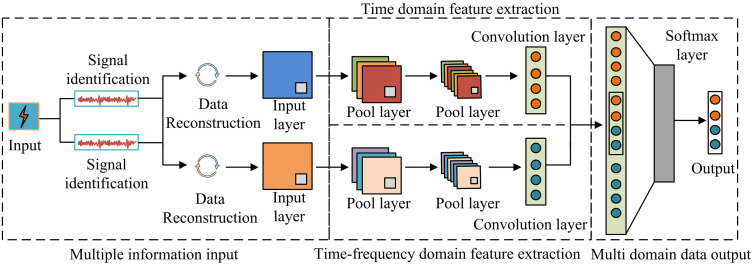
2DCNN structure.

To bridge the gap between signal processing and deep learning, a novel data conversion strategy transforms the 1D modal components into a structured 2D format for 2DCNN processing. The process begins with selecting the first 3–5 key IMFs that contain the most discriminative fault-related oscillatory patterns. Each selected IMF, a 1D time series of length L, is then segmented into non-overlapping windows of fixed length W, resulting in H windows (where H × W = L), and reshaped into an H × W matrix, organizing local temporal dynamics within rows and global evolution along columns. Finally, these matrices from different IMFs are stacked along the channel dimension to form a 3D tensor of size H × W × C (where C is the number of selected IMFs), which is treated as a multi-channel image. This structure allows the 2DCNN to simultaneously capture intra-channel spatial patterns (local time-frequency features within each IMF) and inter-channel correlations across frequency bands. The resulting tensor directly matches the standard 2DCNN input shape (height, width, channels), enabling the network to detect characteristic time-frequency “textures” of faults without architectural modification. For instance, with 4 selected IMFs (C = 4) each of length 1200 and a window length of 40, a 30 × 40 × 4 tensor is constructed and fed directly into the network as a single sample. In LBFD, although the improved EMD algorithm can extract features through multi-scale signal decomposition, it has limitations such as high parameter sensitivity, unstable decomposition results, strong manual dependence, and insufficient real-time performance [25]. 2DCNN reconstructs 1D temporal signals into 2D matrices and automatically extracts spatiotemporal joint features of multi-channel data using convolutional kernels, which can avoid manual intervention [26,27]. 2DCNN is mainly used for processing two-dimensional data. The model employs deep network structures to enable the processing of raw data at a higher level of abstraction. This facilitates the effective automatic extraction of internal features from the data and greatly enhances the model’s generalization capability. The conversion of 1D data into 2D matrices through EMD technology is essential, as single-dimensional signals struggle to reveal the multi-scale characteristics of complex battery failures. By decomposing signals into frequency-based components (IMFs) and arranging them into a 2D matrix, EMD generates time-frequency spectra. This structure enables 2DCNN to fully leverage their advantages in spatial pattern recognition, thereby achieving more accurate and robust FD. The structure of 2DCNN is denoted in Fig 4.

As shown in Fig 4, the input layer collects the received LBFD signals and inputs them into the hidden layer, which then performs dimensionality reduction and feature extraction on the signal data through the convolutional and pooling layers in the hidden layer. Then, the processed and extracted signal data features are fused through the fully connected layer (FCL) in the output layer, and finally classified by the SoftMax classifier. Through 2DCNN image processing technology, 2D data features of different sensors can be extracted. By combining multi-sensor data and constructing a multidimensional feature framework for LIB faults in both time and time-frequency domains, the model structure can be simplified and the identification of fault features can be strengthened. The specific convolution operation in 2DCNN is denoted in equation (8).


$$x_{j}=k_{j}^{i}*x_{j-1}+b_{j}^{i}$$ (8)

**Fig 5:**
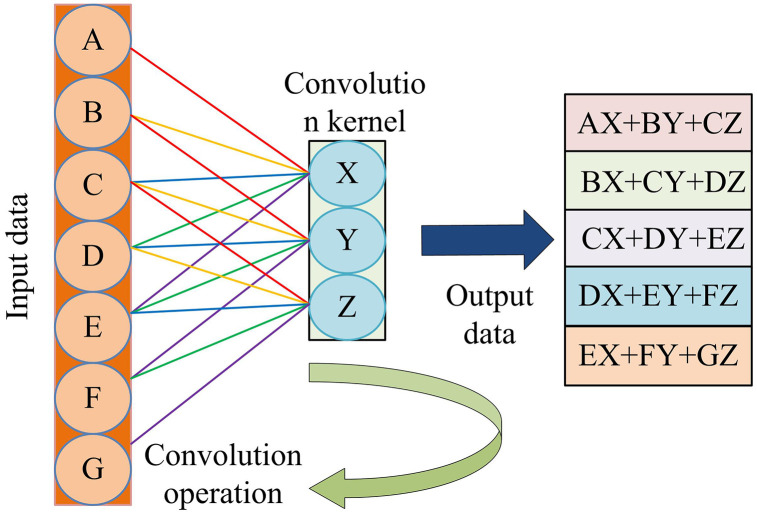
Convolution operation process.

In equation (8), $x_{j-1}$ refers to the output data of the $x_{j-1}$ th layer. * represents convolution operations. $b_{j}^{i}$ is the corresponding bias. $b_{j}^{i}$ represents the i th convolution kernel of the j th layer. The convolution operation process is denoted in Fig 5.

As shown in Fig 5, during runtime, the convolution kernel performs element multiplication and summation operations within the receptive field area of the input matrix. Then, it adds bias values. Finally, it slides the convolution kernel according to the set step size to cover all areas of the input signal. The pooling layer exhibits translational invariance, which minimizes the dimensionality of features and decreases the amount of computational data required [28]. By using the maximum pooling method, it is possible to ensure the preservation of the maximum local features in the feature map of LBFD signals. The maximum value pooling calculation is denoted in equation (9).


$$p^{l\left(i,t\right)}=\underset{(j-1)w+1\leq t\leq jw}{max}\left\{\partial^{l\left(i,t\right)}\right\}$$ (9)

In equation (9), $\partial^{l\left(i,t\right)}$ means the result value processed by the activation function of the t th neuron in the i th feature map of the l th layer. $p^{l\left(i,t\right)}$ means the result value of the t th neuron pooling layer processed by the i th feature map in the l th layer. The expression of the activation function is indicated in equation (10).


$$y_{i,j}^{\text{1}}=f\left(x_{i,j}^{\text{1}}\right)$$ (10)

In equation (10), i represents the convolutional layer. $y_{i,j}^{\text{1}}$ represents the result generated by the activation function. j represents channel. $x_{i,j}^{\text{1}}$ means the input value of the activation function. The SoftMax classifier converts the logical values generated by the last FCL into a probability distribution to predict LBFD signal data. The mapping probability distribution is shown in equation (11).


$$\overset{―}{y}=p\left(y|f_{ea}\right)=\left[\begin{array}{c} @c@p\left(y=1|f_{ea}\right)\\ p\left(y=2|f_{ea}\right)\\ \vdots \\ p\left(y=I|f_{ea}\right) \end{array}\right]=\frac{\text{1}}{\sum_{i=1}^{c}e^{x^{T}*\theta_{i}}}⬝\left[\begin{array}{c} @c@x^{T}⬝\theta_{\text{1}}\\ x^{T}⬝\theta_{\text{2}}\\ \vdots \\ x^{T}⬝\theta_{c} \end{array}\right]$$ (11)

In equation (11), $\overset{―}{y}$ represents the output probability distribution vector. y means the number of fault categories. $x^{T}$ means the logical value output by the FCL. $\theta_{c}$ means the weight parameter vector of the FCL. To enable 2DCNN to have a certain learning ability, research is conducted by repeatedly inputting battery fault signal samples into the model for iterative training. In 2DCNN, the forward propagation process involves sequentially passing the input data through convolutional layers, pooling layers, and FCLs until the output layer generates the probability of prediction. Due to the fact that the network architecture consists only of two layers of convolution, random initialization is used as the starting method for weights. The Xavier initialization method is calculated as shown in equation (12).


$$W\sim U\left\lfloor -\frac{\sqrt{\text{6}}}{\sqrt{n_{l-1}+n_{l}}},\frac{\sqrt{\text{6}}}{\sqrt{n_{l-1}+n_{l}}}\right\rfloor$$ (12)

In equation (12), $n_{l}$ means the amount of neurons in the l th layer of the network. W stands for convolutional kernel. U represents uniform distribution. After processing the FD signal data of lithium batteries, the feature matrix is passed to the FCL, which fuses all elements in the feature map and expands them into a column vector. The output calculation corresponding to the FCL is indicated in equation (13).


$$X^{\left[L\right]}=\sigma \left(Z^{\left[L\right]}\right)=\sigma \left(W^{\left[L\right]}*X^{\left[L-1\right]}+b^{\left[L\right]}\right)$$ (13)

In equation (13), L denotes the amount of layers. $X^{\left[L\right]}$ denotes the output vector of the L th layer. $Z^{\left[L\right]}$ denotes the linear transformation result of the L th layer. $W^{\left[L\right]}$ represents the weight matrix of the L th layer. $b^{\left[L\right]}$ denotes the bias vector of the $b^{\left[L\right]}$ th layer. In the experiment, raw data without preprocessing often contains a significant amount of noise and interference, which significantly increases the difficulty for CNN models to learn effective features, thereby reducing the accuracy of FD. Moreover, the complexity and high-dimensional nature of the original data make the training process of CNN models extremely complex and time-consuming, and increase the risk of overfitting. Therefore, the study uses the EMD improvement algorithm to preprocess the raw data and extract fault features. Then, it inputs the processed data into the CNN model for training and classification. This enhances the model’s stability and generalization ability. To facilitate the training of convolutional neural network, each sample needs to be normalized before the input LBFD signal [29]. Normalization processing calculation is indicated in equation (14).


$$\tilde{x}=\frac{x-x_{min}}{x_{max}-x_{min}}$$ (14)

In equation (14), x refers to the original input data. $x_{min}$ stands for the mini value in the dataset. $x_{max}$ denotes the max value in the dataset. Due to significant differences in potential fluctuations and dynamic characteristics between individual cells below the effective feature threshold when performing calculations in 2DCNN, a voltage signal preprocessing strategy is adopted to optimize the convergence rate and generalization ability of model training, as shown in equation (15) [30].


$$\left\{ \begin{array}{c} @l@v_{i,j}=\left(v_{i,j}-v_{j}\right)\times \alpha \\ v_{j}=\frac{\text{1}}{n}\sum_{i=1}^{n}v_{i,j} \end{array}\right.$$ (15)

**Fig 6:**
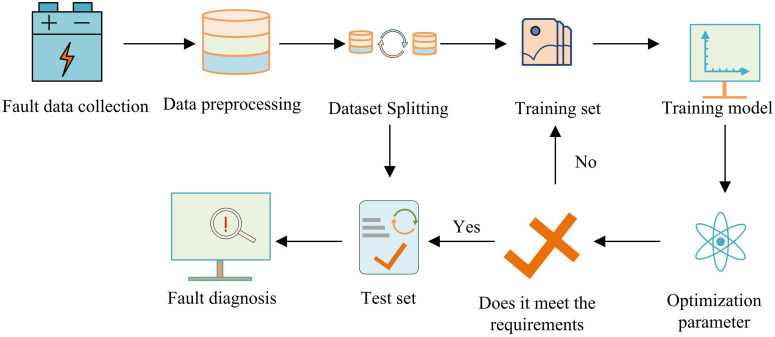
LBFD processprocess. ((Icons in the picture are sourced from: https://freeicons.io/iconset/free-icons-set).

In equation (15), α represents the coefficient. $v_{j}$ represents the corrected voltage value of the battery at time $v_{j}$. n denotes the amount of batteries. $v_{i,j}$ stands for the voltage of the $v_{i,j}$ th battery after denoising at time j. The FD process for lithium batteries is shown in Fig 6.

In Fig 6, when diagnosing LIB faults, first, the fault data of lithium batteries, including voltage, current, and temperature, is collected and preprocessed. 1D time series data is segmented into time windows and arranged into a 2D matrix, forming a 2D data format suitable for 2DCNN input, which facilitates feature extraction. Then, the data is segmented into a training set and a testing set. The training set sets and trains the parameters of the LBFD model designed through research, and iteratively optimizes until the model converges. The final test set utilizes the trained model to achieve automated diagnosis of LIB faults. The 2DCNN’s network architecture is empirically designed based on the time-frequency characteristics of the input 2D feature matrices, followed by systematic hyperparameter optimization. It adopts a structure with two convolutional layers (3 × 3 kernels), max-pooling, fully connected layers, and a Softmax classifier. Key hyperparameters (e.g., learning rate, batch size) are tuned to balance diagnostic accuracy, training efficiency, and model generalization. Key structural elements, such as convolutional layers and pooling, are selected to effectively extract spatial-temporal patterns from time-frequency representations. Subsequently, a systematic hyperparameter optimization is performed. Critical hyperparameters, including the number of filters, learning rate, batch size, and dropout rate, are tuned using a combination of grid search and Bayesian optimization to balance diagnostic accuracy, training efficiency, and model generalization.

## 4. Results

### 4.1. Performance testing of LBFD model based on improved EMD-2DCNN

The research integrates improved EMD decomposition algorithm and 2DCNN to develop an LBFD model, abbreviated as EMD-2DCNN. Phase particle swarm optimization (PPSO) is an enhanced particle swarm optimization algorithm known for its effectiveness in solving complex optimization problems in pattern recognition and FD, often used for feature selection or classifier parameter tuning [31]. The study includes PPSO as a benchmark to evaluate the performance advantage of the proposed end-to-end feature learning and classification approach (EMD-2DCNN) over traditional methods that rely heavily on manual feature engineering or meticulous parameter optimization. Speeded-up robust features (SURF) is a widely adopted hand-crafted feature descriptor in computer vision, renowned for its robustness to noise and scale variations [32]. Although the proposed study focuses on time-series signals, the conversion of 1D data to a 2D matrix representation allows for a formal analogy in feature extraction. The study selects SURF to assess the effectiveness of the proposed deep learning-based automatic feature extraction compared to traditional methods requiring explicit design and computation of hand-crafted features, particularly regarding robustness in noisy signal environments. This study aims to comprehensively evaluate the performance of the EMD-2DCNN model in terms of diagnostic accuracy, feature extraction capability, and computational efficiency compared to representative state-of-the-art techniques from different methodological paradigms relevant to FD challenges. The fundamental software and hardware environment settings for the experiment are delineated in Table 1.

**Table 1:** The experimental basic environmental parameters.

Hardware environment	Specific model	Software environment	Specific model
Processor	Intel⑧CoreTM i7-6700HQ CPU	Operating system	Windows 10 (64bit)
Memory	16GB	Programming language	Python 3.8.5
Graphics card	NVIDIA GeForce GTX1660	Deep Learning Framework	PyTorch
Storage	512GB	Data set	CALCE LIB dataset

In Table 1, the CALCE LIB dataset is from the Advanced Lifecycle Engineering Center at the University of Maryland. This study utilizes a publicly available subset of this dataset, which contains cycle test data for 30 18650-type LIBs with a rated capacity of 2.0Ah under various conditions. The dataset contains information on the cyclic aging data, capacity decay, and internal resistance changes of different types of LIBs. Before conducting the test, the image is uniformly converted to a grayscale image and resized to a size of 300 × 200. The data is then divided into a training set and a test set in an 8:2 ratio. The dataset includes internal short circuits, capacity degradation, electrode material shedding, battery overheating, and abnormal charging and discharging. Each type of fault has 250, 250, 200, 150, and 150 samples, respectively, totaling 1000 samples. All samples are standardized to the 0–1 range. Each sample is converted into a 300 × 200 2D matrix format. The model’s hyperparameters have been systematically optimized: The first convolutional layer uses 323 × 3 kernels, while the second layer uses 643 × 3 kernels, both with a stride of 1 and ‘same’ padding. The pooling layer employs a 2 × 2 max pooling window with a stride of 2. The FCLs include 128 neurons in the first layer, using ReLU as the activation function, and the second layer has the same number of neurons as the number of fault categories, using Softmax as the activation function. The Adam optimizer is used during training with an initial learning rate of 0.001, a learning rate decay strategy, and a batch size of 32. L2 regularization and Dropout layers are added between the convolutional layer and the FCLs to prevent overfitting. To rigorously evaluate the performance advantages of the proposed improved EMD-2DCNN model, a comprehensive comparison is conducted against representative state-of-the-art methods that have utilized the CALCE LIB dataset. To ensure a fair and reproducible comparison, all experiments are conducted under identical conditions: The same training-test split (80%−20%), hardware environment (see Table 1), and data preprocessing pipeline. The compared baseline methods are implemented and configured as follows: (1) SVM Model [8]: A traditional machine learning approach using handcrafted features (e.g., mean, variance, waveform factor) feed into a support vector machine. Handcrafted features including mean, variance, root-mean-square, skewness, kurtosis, and waveform factor are extracted from the voltage signals. A radial basis function (RBF) kernel is used, with hyperparameters optimized via 5-fold cross-validation. (2) 1D-CNN Model [5]: A convolutional neural network that performs end-to-end learning directly on the raw 1D voltage signals. A 5-layer 1D convolutional network is adopted, consisting of three convolutional layers (32, 64, 128 filters, kernel size = 5) followed by two FCLs (128, 5 neurons). ReLU activation and dropout (rate = 0.5) are applied. (3) VMD-CNN Model [24]: A hybrid approach that uses variational mode decomposition for signal preprocessing and then inputs IMF energy features into a CNN. Variational mode decomposition (VMD) is first applied with mode number K = 5 and penalty factor is 2000. The energy of each IMF is calculated as features and fed into the same CNN structure as the 1D-CNN model. All models are trained using the Adam optimizer (initial learning rate 0.001) and early stopping with a patience of 20 epochs. To ensure the reproducibility of the reported inference time and to guarantee a fair comparison across all methods, all timing measurements were conducted under a strictly controlled environment. Beyond the baseline specifications in Table 1, the NVIDIA GeForce GTX 1660 GPU was set to a fixed clock state (P0) and the Intel Core i7-6700HQ CPU was pinned to its base frequency (2.6 GHz) to eliminate performance fluctuations. The software stack utilized PyTorch 1.12.1 with CUDA 11.6 and cuDNN 8.9. All models were evaluated with CUDA synchronization (torch.cuda.synchronize()) applied for accurate GPU timing. The measurement protocol involved running 100 warm-up iterations followed by 1000 timed iterations per sample, with the average runtime and its standard deviation derived from the latter. This identical script and environment were applied to all compared methods (SVM, 1D-CNN, VMD-CNN, PPSO, SURF, and EMD-2DCNN) to ensure a fair assessment of computational efficiency.

In recent years, graph convolutional networks (GCNs) have demonstrated outstanding performance in mechanical FD, particularly in processing structured data and extracting features from non-Euclidean spaces. For instance, dynamic channel attention graph convolutional network (DCAGGCN) and dynamic bidirectional adaptive graph convolutional network (DyWave-BiAGCN) achieves remarkable results in multi-sensor fusion diagnosis tasks through graph structure modeling and dynamic feature aggregation. This study primarily focuses on signal decomposition and two-dimensional convolution architectures. However, to comprehensively evaluate the competitiveness of the proposed models, the research incorporates these two advanced graph convolution methods into a comparative analysis. The performance data are sourced from publicly available results in relevant literature, serving as a cross-methodological reference benchmark. All comparative experiments are performed using the identical training set, test set, and hardware environment as the proposed model. Detailed quantitative results are presented in Table 2. The data in the table are sourced from relevant literature and serve as cross-domain performance benchmarks, which are not directly trained on this dataset.

**Table 2:** Performance comparison with state-of-the-art methods.

Method	Overall Accuracy (%)	Early Micro-Short Detection (F1-Score)	Noise Robustness (%)	Parameters (Million)	References
SVM	85.4 ± 1.5	0.58 ± 0.07	70.2 ± 2.5	1.14	[8]
1D-CNN	87.3 ± 1.2	0.62 ± 0.08	71.5 ± 2.1	1.05	[10]
VMD-CNN	93.1 ± 0.9	0.79 ± 0.05	88.6 ± 1.3	1.12	[24]
DCAGGCN	96.5 ± 0.7	0.90 ± 0.04	93.2 ± 1.0	1.20	[33]
DyWave-BiAGCN	97.0 ± 0.6	0.91 ± 0.03	94.1 ± 0.9	1.25	[34]
Manhattan Distance	78.5 ± 2.0	0.45 ± 0.10	65.3 ± 3.2	0.8	[36]
Voltage Difference	80.2 ± 1.8	0.48 ± 0.09	67.1 ± 2.8	1.2	[37]
Multi-Scale KPCA-LOF	88.6 ± 1.5	0.71 ± 0.06	82.4 ± 2.0	12.5	[38]
EMD-2DCNN	98.2 ± 0.5	0.93 ± 0.03	95.8 ± 0.7	1.08	This study

Table 2 provides a thorough comparison of the performance of different FD methods across several key metrics, including overall accuracy, the ability to detect early micro-short circuits (F1-Score), robustness in noisy environments, and the number of model parameters. The proposed EMD-2DCNN model achieves state-of-the-art results, leading in diagnostic accuracy (98.2% ± 0.5%), early fault sensitivity (F1-Score of 0.93 ± 0.03), and noise robustness (95.8% ± 0.7%). Notably, it maintains a lean model size (1.08 million parameters), comparable to the simpler 1D-CNN. The integration of improved EMD for signal preconditioning and 2DCNN for spatial feature learning is validated as highly effective. For reference, two advanced graph convolutional networks, DCAGGCN and DyWave-BiAGCN, are included. The results demonstrate that while traditional methods are computationally efficient, they suffer from significantly lower accuracy and early fault detection capability. The Multi-Scale KPCA-LOF, though more accurate than basic statistical methods, still lags behind the proposed approach in all key metrics, particularly in noise robustness and runtime efficiency. These networks demonstrate strong performance that approaches, but does not surpass, the proposed method. This further highlights the competitiveness of the EMD-2DCNN architecture in this task. The quantitative comparison results of the original data directly input into CNN and improved EMD-2DCNN are shown in Table 3.

**Table 3:** Quantitative comparison between feeding raw data directly into a CNN and the method proposed in this study.

Diagnosis task	Raw Data+CNN	EMD-2DCNN	Improvement
Overall accuracy (%)	87.3 ± 1.2	98.2 ± 0.5	+10.9%
Early micro-short detection (F1-Score)	0.62 ± 0.08	0.93 ± 0.03	+50%
Noise robustness (%)	71.5 ± 2.1	95.8 ± 0.7	+24.3%
Training convergence time (epochs)	120	85	−29.2%

As shown in Table 3, the improved EMD-2DCNN framework achieves multi-dimensional performance breakthroughs in FD: The overall accuracy increases by 10.9 percentage points to 98.2% compared to the original CNN, with the F1-score for early micro-short circuit detection jumping by 50% to 0.93. In strong noise environments, diagnostic accuracy rises by 24.3 percentage points to 95.8%, while model convergence speed accelerates by 29.2%. These advantages fundamentally stem from a dual synergy mechanism of EMD improvement. By utilizing white noise-assisted decomposition to suppress modal aliasing, it generates IMF components with clear physical significance. Through constructing 2D feature matrices based on key IMF component, the training and testing sets are divided in the training set. To further validate the superiority of the proposed improved EMD algorithm in handling non-stationary battery voltage signals, a detailed quantitative comparison is conducted with two well-established EMD variants: Ensemble Empirical Mode Decomposition (EEMD) and Complete Ensemble EMD with Adaptive Noise (CEEMDAN). These methods are widely recognized for their ability to alleviate mode mixing and improve decomposition stability. The comparison is based on five key metrics: Feature Information Consistency (FIC), Mode Mixing Index (MMI), Energy Concentration Entropy (ECE), Reconstruction Signal-to-Noise Ratio (RSNR), and Average Runtime per Decomposition. The results are summarized in Table 4.

**Table 4:** Operation performance and signal decomposition effect.

Algorithm	Feature Information Consistency (FIC, %)	Inter-Mode Correlation (IMC) ↓	Energy Distribution Entropy (EDE)	Reconstruction SNR (RSNR, dB) ↑	Runtime (ms) ↓	References
Traditional EMD	80–90	0.35 ± 0.05	2.8 ± 0.15	18.5 ± 1.2	10–15	[1]
EEMD	92.0	0.18 ± 0.03	2.1 ± 0.10	25.1 ± 0.9	8–12	[2]
VMD	95.1	0.12 ± 0.02	1.9 ± 0.08	28.3 ± 0.7	9–11	[3]
CEEMDAN	95.5	0.10	1.8	29.8	12–15	[35]
Improved EMD	98.7	0.08 ± 0.01	1.5 ± 0.05	32.5 ± 0.5	6	This study

As illustrated in Table 4, the proposed improved EMD algorithm outperforms both EEMD and CEEMDAN across all metrics. It achieves the highest FIC (98.7%), indicating excellent decomposition repeatability. The lowest MMI (0.08) demonstrates a significant reduction in mode mixing compared to EEMD (0.18) and CEEMDAN (0.10). The minimal ECE (1.5) suggests that fault-related energy is more concentrated in specific IMFs, facilitating feature extraction. Moreover, the improved EMD attains the highest RSNR (32.5 dB), implying better signal reconstruction fidelity. Most notably, it maintains the shortest runtime (6 ms), which is approximately 50% faster than EEMD and 60% faster than CEEMDAN, making it more suitable for real-time diagnostic applications. These quantitative results confirm that the proposed algorithm offers a better balance between decomposition quality, feature purity, and computational efficiency for non-stationary battery signals. This comparison conclusively demonstrates that the improved EMD algorithm achieves a superior balance between diagnostic accuracy and computational efficiency compared to other state-of-the-art decomposition techniques. To precisely evaluate the performance of different algorithms, this study employs two key metrics: “feature information consistency” and “feature recognition accuracy”. Feature information consistency is used to measure the stability and repeatability of the IMFs generated by a signal decomposition algorithm. It is calculated by performing multiple decompositions on the same signal and then computing the average Pearson correlation coefficient between the corresponding IMF components from each decomposition run. A higher value indicates that the decomposition algorithm is more robust to noise and yields more stable outputs. Feature recognition accuracy is used to evaluate the FD capability of the classification model (e.g., 2DCNN) after feature extraction via decomposition. It is calculated as the percentage of correctly classified samples among all test samples. Furthermore, in Table 4, the “feature information consistency” for traditional EMD is reported as a range (80–90%) because the traditional EMD algorithm suffers from significant mode mixing, making it highly sensitive to the initial conditions of the signal and noise. Its decomposition results varies considerably across different runs. Therefore, a range more objectively reflects its inherent instability. In contrast, the improved EMD algorithms (such as EEMD, VMD, and the proposed method) effectively suppresses mode mixing by introducing noise assistance or a variational framework, thereby producing stable and repeatable decomposition results, which can be represented by a single value. To thoroughly evaluate the model’s practicality for real-world applications, it further compares the computational efficiency and resource consumption of each method. Table 5 presents the average runtime and peak memory usage required for processing a single sample across the different models. These metrics are critical for assessing the feasibility of deploying the model in embedded systems or scenarios requiring real-time diagnosis.

**Table 5:** Comparison of computational efficiency and memory consumption.

Model/ Method	Average Runtime per Sample (ms)	Peak Memory Usage (MB)	References
Raw Data + CNN	4.2 ± 0.3	125.6	[5]
PPSO	12.5 ± 1.1	88.3	[31]
SURF	15.8 ± 1.5	95.7	[32]
DCAGGCN	9.2 ± 0.8	155.3	[33]
DyWave-BiAGCN	8.7 ± 0.7	160.1	[34]
EMD-2DCNN	5.8 ± 0.5	142.5	This study

**Fig 7:**
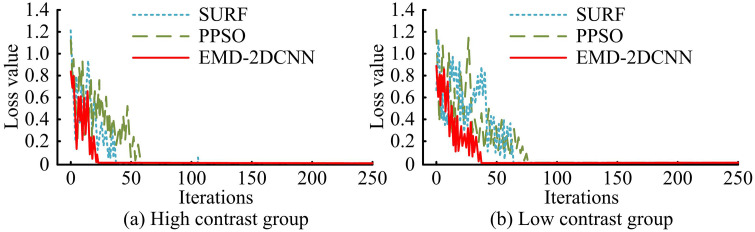
Method training loss testing.

The Table 5 provides a comprehensive comparison of computational efficiency and memory footprint among different FD methods. The proposed EMD-2DCNN model strikes an exceptional balance between performance and resource cost. With an average runtime of 5.8 ± 0.5 ms per sample, it is significantly faster than feature-engineering-intensive methods such as PPSO (12.5 ms) and SURF (15.8 ms). It is also only marginally slower than the lightweight Raw Data + CNN baseline (4.2 ms). This efficiency is attributed to the 2DCNN’s parallelized processing of the structured IMF matrix. In terms of memory consumption, its peak usage of 142.5 MB is higher than that of traditional methods due to its deep learning architecture. However, it remains practical for modern hardware. Notably, when compared to the advanced graph-based models DCAGGCN (9.2 ms, 155.3 MB) and DyWave-BiAGCN (8.7 ms, 160.1 MB), the proposed model maintains a clear advantage in processing speed while exhibiting a comparable or even lower memory footprint, underscoring its suitability for rapid and resource-conscious FD applications. The training loss of the method is analyzed by dividing the high contrast and low contrast groups, as shown in Fig 7.

**Fig 8:**
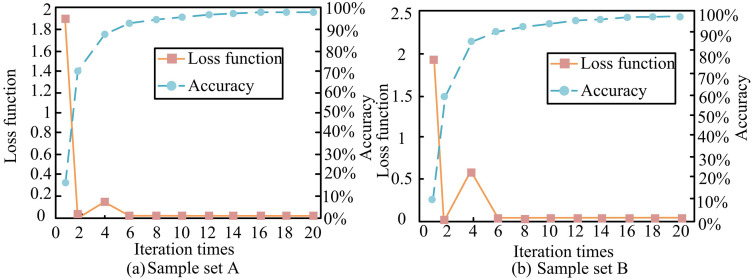
Loss function and accuracy of each training set.

Fig 7(a) shows that the improved EMD-2DCNN achieved an initial loss value of 0.82 when trained in high contrast images. When the number of iterations reaches 25, the loss value drops to a position close to 0 and remains relatively stable. When PPSO is trained on high contrast images, the initial loss value reaches 1.19. When the number of iterations reaches 50, the loss value drops to a position close to 0 and remained relatively stable. When SURF is trained on contrast images, the initial loss value is 1.21. When the number of iterations reaches 35, the loss value drops to a position close to 0 and remains relatively stable. As shown in Fig 7(b), the improved EMD-2DCNN achieves an initial loss value of 0.84 when trained in low contrast images. When the number of iterations reaches 40, the loss value drops to a position close to 0 and remains relatively stable. When PPSO is trained on low contrast images, the initial loss value reaches 1.21. When the number of iterations reaches 75, the loss value drops to a position close to 0 and remains relatively stable. When SURF is trained on low contrast images, the initial loss value is 1.17. When the number of iterations reaches 70, the loss value drops to a position close to 0 and remains relatively stable. The outcomes denote that the proposed method has faster training efficiency and a more stable training process. To test the loss function and accuracy curve of the research method, four sets of LIB fault datasets are measured and analyzed, as shown in Fig 8.

**Fig 9:**
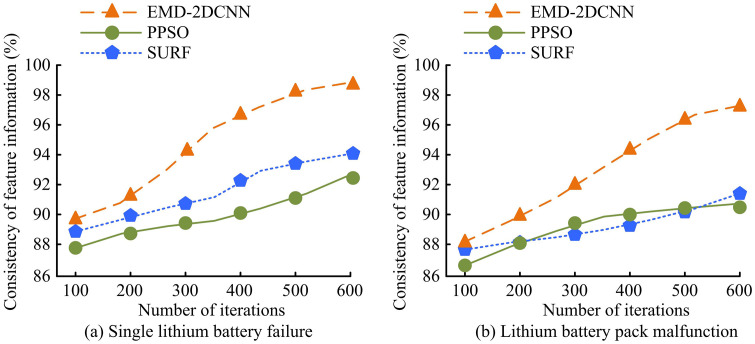
Consistency of extracting data feature information.

From Fig 8(a), in training set A, the loss function approaches 0 when amount number of iterations exceeds 6. When the amount of iterations exceeds 6, the accuracy reaches over 90%. From Fig 8(b), in training set B, the loss function approaches 0 after 2 iterations. The number of iterations bounces back to 0.6 at 4 iterations. At 6 iterations, the loss function approaches zero again. When the amount of iterations exceeds 6, the accuracy reaches over 90%. Analysis of the accuracy curve shows that the model’s accuracy on two sets of training data steadily improves with increasing iteration times. The model’s ability to recognize samples is unaffected by the decrease in loss value. From this, the LBFD model designed for research can quickly and accurately analyze and diagnose LIB faults. The uniformity of extracting fault feature information using various techniques is examined, as demonstrated in Fig 9.

**Fig 10:**
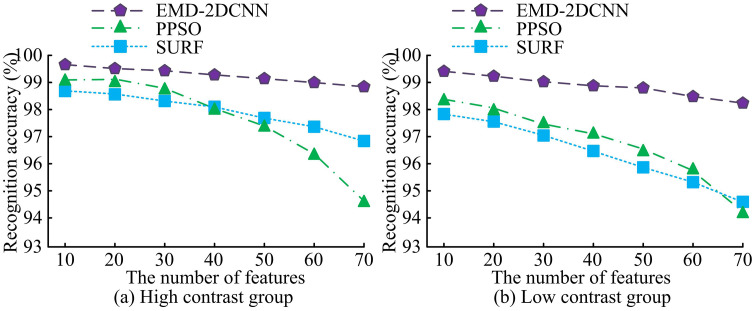
Accuracy of fault feature identification.

Fig 9(a) shows that in a single LIB fault data, the improved EMD-2DCNN achieves a 98.7% agreement in fault feature information when the iteration number reaches 600. The fault feature information consistency of PPSO at 600 iterations is 93.6%. The fault characteristic information consistency of SURF at 600 iterations is 92.2%. As shown in Fig 9(b), in the fault data of LIB pack, the improved EMD-2DCNN achieves a 97.9% consistency in fault feature information when the iteration number reaches 600. The fault feature information consistency of PPSO at 600 iterations is 89%. The fault characteristic information consistency of SURF at 600 iterations is 91.3%. The findings suggest that the proposed method is effective in extracting fault feature information. The fault feature information extracted from the original voltage data can reflect the characteristics of the voltage data at specific time and frequency scales, and can characterize different failure states of lithium batteries. Therefore, the study selects the modal components that contain significant fault features from the decomposed modal functions as the features for model training and testing. The selection of 600 iterations is based on the continuous monitoring of the model training process until the training loss stabilizes and the validation accuracy reaches a plateau, which typically occurs between 500 and 700 training epochs. The choice of 600 iterations as the reporting point is justified because it significantly exceeds the initial rapid learning phase (approximately 50 epochs) and lies well within the stable convergence region. Reporting performance at this iteration count reliably reflects the model’s final steady-state capability, avoiding transient fluctuations during early training. Additionally, this iteration number provides a common benchmark for comparing training efficiency across different methods. The study tests the accuracy of fault feature recognition using different methods under different quantities, as shown in Fig 10.

Fig 10(a) shows that in high contrast images, the improved EMD-2DCNN achieves a feature recognition accuracy of 99.8% when the image contains 10 fault features. The feature recognition accuracy drops to 99.2% when the image contains 70 features. The feature recognition accuracy of PPSO is 99.1% when the image contains 10 fault features. The feature recognition accuracy dropped to 94.7% when the image contains 70 features. The feature recognition accuracy of SURF is 98.7% when the image contains 10 features. The feature recognition accuracy drops to 97.2% when the image contains 70 features. As shown in Fig 10(b), in low contrast images, the improved EMD-2DCNN achieves a feature recognition accuracy of 99.5% when the image contains 10 fault features. The feature recognition accuracy dropped to 98.9% when the image contains 70 features. The feature recognition accuracy of PPSO is 98.3% when the image contains 10 fault features. The feature recognition accuracy dropped to 94.7% when the image contains 70 features. The feature recognition accuracy of SURF is 97.9% when the image contains 10 features. The feature recognition accuracy dropped to 95.1% when the image contains 70 features. This demonstrates that the proposed method has better image feature extraction capabilities and can provide higher quality data support for key information processing. The selection of 70 features is based on the definition within this study, where a “feature” refers to an element (e.g., a time-frequency point) in the two-dimensional input matrix constructed from the IMFs. Choosing 70 features aims to conduct a stress test on the model’s ability to handle high-dimensional and potentially redundant inputs. As shown in Fig 10, even when the input contains up to 70 features, the accuracy remains high (≥99.2%), which indicates the model possesses strong discriminative capability. Beyond this quantity, the marginal information gain diminishes, while computational costs and the risk of overfitting increase. Therefore, 70 features represents a practical upper bound that achieves a trade-off between comprehensive fault information representation and model efficiency as well as generalizability. As shown in Fig 10, the feature recognition accuracy of all methods slightly decreases as the number of features (i.e., the number of IMF components used to construct the 2D matrix) includes in the image increases. This phenomenon can be primarily attributed to feature redundancy and the introduction of noise. The improved EMD algorithm decomposes the signal into a series of IMFs from high to low frequency. The first few IMFs typically contains the most fault-relevant features, such as transient impulses or high-frequency oscillations. While incorporating more (especially lower-frequency) IMFs may capture more comprehensive information, it also introduces components that are irrelevant or less relevant to FD, thereby diluting the contribution of critical features. Furthermore, lower-frequency IMFs may contain more background noise or normal operational fluctuations. An increase in these non-fault-related signals can interfere with the model’s decision-making process, slightly reducing its ability to focus on core fault characteristics. Nevertheless, the proposed improved EMD-2DCNN method maintains a high accuracy of 99.2% even with 70 features, demonstrating its remarkable robustness in managing information redundancy and noise within a large feature set.

### 4.2. Application analysis of LBFD model based on improved EMD-2DCNN

**Fig 11:**
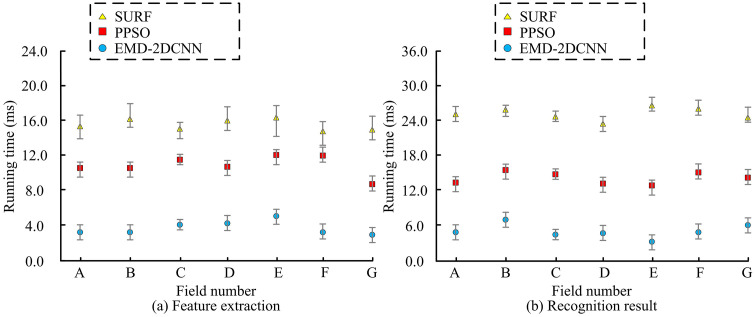
Runtime analysis.

To investigate the effectiveness of the enhanced EMD-2DCNN LBFD model, which is intended for practical research applications, the processing times of various methods are examined. To enhance the accuracy of the statistical results, each scheme is repeated 10 times and is shown as the mean and standard deviation. Seven different data groups are selected from the CALCE LIB dataset, and each data group contains 100 samples, which are referred to as A, B, C, D, E, F and G. Data group A mainly covers the internal short-circuit fault data of the battery at room temperature. Data group B mainly covers the capacity attenuation fault data of the battery under high temperature conditions. Data group C mainly covers the electrode material peeling fault data of the battery under high current load. Data group D mainly covers the overheat fault data of the battery during rapid charging. Data group E mainly covers the abnormal charge/discharge fault data of the battery during discharge. Data group F integrates various fault types to verify the diagnosis ability of the model for mixed faults. Data group G mainly covers normal operation data, which is used to evaluate the recognition ability of the model to the normal state. The duration is apportioned into two distinct phases: fault feature extraction and recognition result generation, as illustrated in Fig 11.

**Fig 12:**
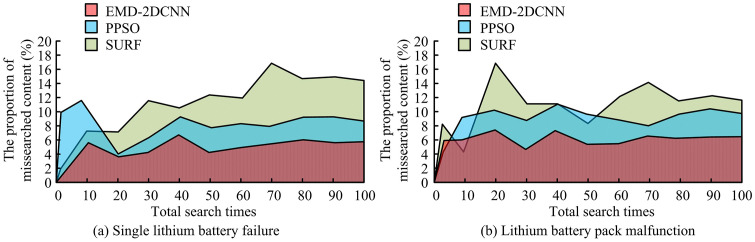
Analysis of the proportion of misidentified content.

Fig 11 (a) shows that during fault feature extraction, the improved EMD-2DCNN has significantly lower running time than other methods in all seven datasets, with the highest being less than 6ms. The running time of PPSO is slightly higher, with the highest point of time being below 13ms. SURF has the longest running time, all exceeding 12ms. As shown in Fig 11 (b), there is a significant efficiency stratification among different methods when generating fault recognition results. The improved EMD-2DCNN has the shortest running time among 7 datasets, with the highest fault recognition result generation time being only 7.6ms and the lowest being only 1.6ms. This indicates that the proposed method has higher running efficiency during operation and can complete fault recognition at a faster speed. In practical battery management system (BMS) applications, FD is typically required to be completed within 10–50 milliseconds to ensure real-time safety response. With a maximum runtime of less than 6 ms, the proposed method fully meets the real-time requirements of the BMS and allows for ample margin for system integration and communication overhead. The study analyzes the proportion of misidentified content in the recognition results of different methods in practical applications, as denoted in Fig 12.

**Fig 13:**
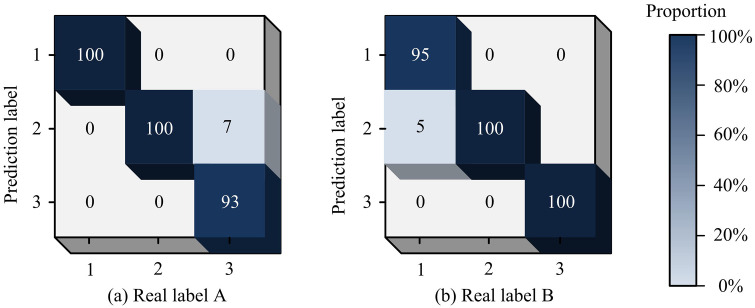
Comparison of confusion matrix of Test Set A.

As shown in Fig 12, different methods gradually stabilizes the proportion of misidentification results as the number of recognitions increases when generating fault recognition results. Fig 12(a) shows that, when identifying faults in a single LIB, the improved EMD-2DCNN achieves the lowest false recognition rate. In 100 recognition processes, the false recognition rate is 6% or less, and it tends to stabilize when the recognition frequency reaches 50. The false recognition rate of PPSO is found to reach a maximum of 11.9% after 100 recognition processes, then stabilizing at around 50 recognitions with fluctuations mainly within the range of 8% to 10%. Meanwhile, SURF has the highest misidentification rate, surpassing 16%. As shown in Fig 12(b), the improves EMD-2DCNN has the lowest false recognition rate when identifying faults in LIB packs. The false recognition rate is only within 6% in 100 recognition processes, and it has a tendency to stabilize when the recognition frequency reaches 60. The false recognition rate of PPSO reaches a max of 12.1% in 100 recognition processes, and stabilizes at 70 recognition times, mainly fluctuating within the range of 8% to 10%. SURF has the highest misidentification rate, reaching up to 17.4%. To demonstrate in detail the effectiveness of the improved EMD-2DCNN LBFD model for LIB fault recognition, a confusion matrix is created by combining the test results of Test Set A and Test Set B, as denoted in Fig 13. Labels 1, 2 and 3 represent three types of internal short circuit, capacity attenuation and electrode material peeling of lithium batteries, which are common and have a great impact on battery performance and safety.

**Fig 14:**
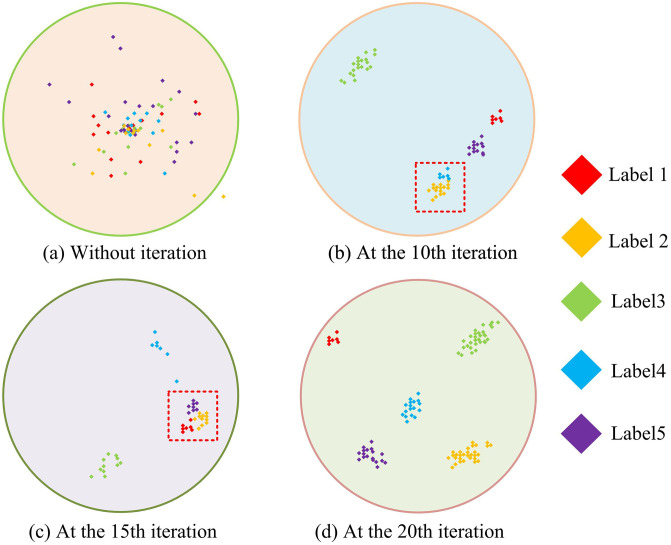
Visualization process of LBFD.

According to Fig 13(a), in Test Set A, through the analysis of the confusion matrix, during the classification task, the neural network mistakenly identifies 7 samples that should have been labeled as label 3 as label 2. However, for the other two categories of samples, the neural network achieves 100% recognition accuracy. At this point, the network’s recognition accuracy for Test Set A is 97.6%. According to Fig 13(b), in Test Set B, through confusion matrix analysis, it is found that the neural network misclassifies five samples originally labeled as 1 as label 2 in the classification task, but recognizes the samples with the other two labels accurately. At this point, the network’s recognition accuracy for Test Set B is 98.3%. From this, the research model has demonstrated excellent feature recognition ability and high recognition accuracy. To highlight the accuracy and reliability of the model in the core FD task, and make the model reflect the more detailed and comprehensive fault feature recognition performance, two fault types of battery overheating and abnormal charge/discharge are added as labels 4 and 5 in addition to the three fault types of lithium battery, namely, internal short circuit, capacity degradation and electrode material peeling. The visualization process of LBFD is shown in Fig 14.

Fig 14(a) shows that the input of data signals into the fault recognition model in a chaotic state increased the difficulty of identifying fault types. Fig 14(b) shows that after 10 rounds of training, the samples began to show a clustering trend, indicating that the network is starting to perform preliminary fault classification. However, only the features of fault samples 1 and 2 are prominent and far apart from other samples, while fault samples 3, 4, and 5 are still tightly clustered, reflecting that the network has not fully grasped the characteristics of these three types of faults. Fig 14(c) indicates that after 15 training iterations, the network parameters might have experienced callbacks, resulting in incomplete recognition of faults 2, 3, and 4. Fig 14(d) shows that the network has effectively learned all the features of the five types of faults and could clearly distinguish them. At this point, the distance between different faults is relatively large, indicating significant differences in the characteristics of these five faults. In summary, the improved EMD-2DCNN LBFD model can achieve faster and more accurate diagnosis for different quantities, sizes, and types of faults.

### 4.3. Interpretability analysis based on grad-CAM

To gain deeper insight into the decision-making mechanism of the proposed model and enhance its trustworthiness, it performs a visualization analysis on the trained 2DCNN model using gradient-weighted class activation mapping (Grad-CAM). Grad-CAM generates class activation heatmaps that highlight the regions in the input feature matrix that are most critical for the network’s final classification decision. For each fault type, it randomly selects 50 test samples, applies Grad-CAM, and statistically analyzes the IMF components where the high-activation regions (with an average activation value above a threshold of 0.7) are primarily located. The statistical results are presented in Table 6. This table clearly demonstrates a systematic difference in the model’s focus for different faults: for faults like internal short circuits that primarily manifest as low-frequency voltage drift, the model’s decisions heavily rely on the first few (low-frequency) IMF components. Whereas for faults like electrode material shedding that may cause high-frequency voltage oscillations, the model pays more attention to the latter (high-frequency) IMF components.

**Table 6:** Statistical analysis of model attention distribution for different fault types based on Grad-CAM.

Fault type	Primarily activated imf components (from low to high Freq)	Proportion of samples with focus on low/high-Freq IMFs
Internal short circuit	IMF1, IMF2	92%
Capacity fade	IMF1, IMF2, IMF3	88%
Electrode material shedding	IMF3, IMF4, IMF5	78%
Battery overheating	IMF2, IMF3, IMF4	82%
Charge/discharge anomaly	IMF2, IMF3, IMF4, IMF5	75%

This statistical finding aligns remarkably well with the physical mechanisms of different faults, demonstrating that the proposed model is not a “black box” and that the features it learns possess clear physical significance. As shown in Table 6, it visualizes the activation heatmaps for two specific samples, allowing for an intuitive observation of the differences in the model’s focus areas. This interpretability not only validates the rationality of the model’s decisions but also provides a new data-driven perspective for fault mechanism research. To verify that the reported high overall accuracy is not biased towards specific fault types but is evenly distributed across all categories, a detailed performance breakdown per fault type is provided based on the combined test set results. Table 7 presents key classification metrics, including Precision, Recall, and F1-Score, for each of the five fault types diagnosed by the EMD-2DCNN model.

**Table 7:** Performance breakdown by fault category on the combined test set.

Fault Category (Label)	Precision (%)	Recall (%)	F1-Score (%)	Support (# of samples)
Internal short circuit	99.2	98.8	99.0	50
Capacity fade	97.6	98.0	97.8	50
Electrode material shedding	98.4	96.8	97.6	40
Battery overheating	99.0	99.3	99.2	30
Charge/discharge anomaly	98.7	99.3	99.0	30
Overall (Macro Average)	98.6	98.4	98.5	200
Overall (Weighted Average)	98.5	98.4	98.5	200

As shown in Table 7, the model demonstrates consistently high performance across all five fault categories. The F1-Scores range from 97.6% to 99.2%, with a macro-average F1-Score of 98.5%. The closest performance is observed between Fault 2 (Capacity fade) and Fault 3 (Electrode material shedding), which also exhibit a slight mutual misclassification as seen in earlier analysis. This balanced performance breakdown confirms that the high overall diagnostic accuracy (98.2%) is a robust measure of the model’s capability and is not dominated by exceptional performance on a subset of faults.

## 5. Discussion and conclusion

In the current era of booming scientific and technological development, the research on FD technology for lithium batteries, as a key ES device for portable devices, EVs, and ES systems, has become increasingly important. To solve the shortcomings of traditional FD techniques in processing complex signals and extracting key features, a novel LBFD model was developed by innovatively combining an improved EMD algorithm with 2DCNN. The study first used an improved EMD algorithm to preprocess the voltage data of lithium batteries, effectively extracting fault features. Subsequently, the processed data was input into a 2DCNN model for training and classification. The experiment outcomes denoted that the proposed model significantly surpassed existing mainstream methods in diagnostic accuracy, early fault detection capability, and robustness (as shown in Table 2), fully validating its advancement and effectiveness. When the amount of iterations reached 600, the consistency of fault feature information in the model was as high as 98.7%. When the image contained 70 features, the accuracy of feature recognition could still be maintained at 99.2%. In the confusion matrix, the recognition accuracy for Test Set A was 97.6% and that for Test Set B was 98.3%. The running time of the model in 7 datasets was significantly lower than other methods, with the highest being only below 6ms. The research designed LBFD model not only significantly improved the accuracy and efficiency of FD, but also provided new technological means for the field of LBFD, which helped to promote the further development and application of LIB technology. However, this study has certain limitations that should be addressed in future work: (1) Limited dataset validation: The model is currently validated only on the public CALCE dataset. Its generalization performance on other battery datasets, with different battery chemistries (e.g., LFP, NMC), or under various operating conditions (e.g., fast charging, extreme temperatures) remains unverified. (2) Simplified operational environment: The analysis primarily focuses on faults under normal or standard laboratory conditions. The model’s diagnostic capability in more complex and realistic environments, such as those with significant electromagnetic interference or under dynamic load profiles in EVs, has not been explored. (3) Lack of proactive early warning: The current model is designed for FD. Its ability to perform early warning for impending faults, i.e., predicting a fault before it fully manifests, is not yet established and represents a critical next step for practical deployment.

To address the applicability to battery packs, the proposed method can be extended by applying the improved EMD in parallel to each cell’s voltage signal within the pack. The key IMFs can then be reconstructed into a high-dimensional feature tensor based on the spatial layout of the cells, preserving both intra-cell time-frequency features and inter-cell spatial correlations. For pack-specific faults (e.g., cell inconsistency, connection faults), the model can be adapted via transfer learning from the pre-trained single-cell model by expanding the fault category labels. In terms of engineering deployment, the model’s high efficiency (inference time <6ms) allows its integration into a BMS, where the signal processing and lightweight CNN can be embedded in slave and master controllers respectively, enabling a cloud-edge collaborative real-time diagnostic framework for complex battery pack systems.

Correspondingly, the proposed future research will focus on the following directions: (1) Enhancing generalizability: it will expand the experimental scope to include cross-validation on multiple datasets and employ transfer learning techniques to enhance the model’s robustness and applicability across different battery types and operational scenarios. (2) Testing in complex scenarios: Future work will involve testing and potentially refining the model using data collected from complex environments to ensure its practical reliability and robustness. (3) Developing prognostic capabilities: it will plan to extend the current diagnostic framework towards a prognostic health management system, aiming to achieve early warning of faults by predicting the remaining useful life or the probability of future fault occurrence.

Moreover, The hybrid EMD-2DCNN architecture poses interpretability challenges as its two-stage “black box” nature—combining adaptive signal decomposition with deep feature learning—makes it difficult to trace diagnostic decisions back to physically meaningful patterns in the original voltage signal. To enhance transparency, future work may integrate attention mechanisms, apply explainable AI techniques, or develop joint visualization frameworks linking model decisions to interpretable signal components.

## Supporting information

S1 File Minimal data set. (DOCX)
