# Supplementary material for: Lithium battery fault diagnosis by integrating improved EMD decomposition algorithm and 2DCNN
Source: PLoS One. 2026 Mar 17;21(3):e0344847. doi: 10.1371/journal.pone.0344847 (PMC12994844; doi:10.1371/journal.pone.0344847)
Supplement: S1 File — (DOCX) [file pone.0344847.s001.docx]

\\10.4.10.66\Newgen\AutoComp\MikTex\New math Tool**The data in Figure 7**

| Iterations | High contrast group(Loss value) | | |
| --- | --- | --- | --- |
|  | EMD-2DCNN | PPSO | SURF |
| 0 | 0.82 | 1.19 | 1.21 |
| 50 | 0.04 | 0.06 | 0.07 |
| 100 | 0.03 | 0.04 | 0.05 |
| 150 | 0.01 | 0.03 | 0.04 |
| 200 | 0.01 | 0.01 | 0.03 |
| 250 | 0.01 | 0.01 | 0.01 |
| Iterations | Low contrast group(Loss value) | | |
|  | EMD-2DCNN | PPSO | SSD |
| 0 | 0.84 | 1.21 | 1.17 |
| 50 | 0.04 | 0.07 | 0.06 |
| 100 | 0.03 | 0.05 | 0.04 |
| 150 | 0.01 | 0.04 | 0.03 |
| 200 | 0.01 | 0.02 | 0.01 |
| 250 | 0.01 | 0.01 | 0.01 |

**The data in Figure 8**

| Iterations | Sample set A | | Sample set B | |
| --- | --- | --- | --- | --- |
|  | Loss function | Accuracy (%) | Loss function | Accuracy(%) |
| 0 | 1.9 | 16 | 1.9 | 9 |
| 2 | 0.03 | 76 | 0.02 | 68 |
| 4 | 0.18 | 88 | 0.6 | 86 |
| 6 | 0.02 | 91 | 0.03 | 89 |
| 8 | 0.02 | 94 | 0.02 | 92 |
| 10 | 0.02 | 95 | 0.02 | 94 |
| 12 | 0.02 | 96 | 0.02 | 95 |
| 14 | 0.02 | 97 | 0.02 | 97 |
| 16 | 0.02 | 98 | 0.02 | 97 |
| 18 | 0.02 | 98 | 0.02 | 97 |
| 20 | 0.02 | 98 | 0.02 | 97 |

**The data in Figure 9**

| Iterations | Single lithium battery failure(Consistency of feature information (%)) | | |
| --- | --- | --- | --- |
|  | EMD-2DCNN | PPSO | SURF |
| 100 | 89.8 | 89 | 87.6 |
| 200 | 91.7 | 89.9 | 88.7 |
| 300 | 94.3 | 90.8 | 89.5 |
| 400 | 96.6 | 92 | 90.1 |
| 500 | 98 | 93.1 | 91 |
| 600 | 98.7 | 93.6 | 92.2 |
| Iterations | Lithium battery pack malfunction(Consistency of feature information (%)) | | |
|  | EMD-2DCNN | PPSO | SSD |
| 100 | 88.1 | 86.8 | 87.8 |
| 200 | 89.9 | 88.1 | 88.2 |
| 300 | 92 | 88. | 88.6 |
| 400 | 94.3 | 88.4 | 89.4 |
| 500 | 96.2 | 88.7 | 90.2 |
| 600 | 97.9 | 89 | 91.3 |

**The data in Figure 10**

| The number of features | High contrast group(Recognition accuracy (%)) | | |
| --- | --- | --- | --- |
|  | EMD-2DCNN | PPSO | SURF |
| 10 | 99.8 | 99.1 | 98.7 |
| 20 | 99.7 | 99 | 98.5 |
| 30 | 99.6 | 98.7 | 98.3 |
| 40 | 99.5 | 98.1 | 98 |
| 50 | 99.4 | 97.4 | 97.8 |
| 60 | 99.3 | 96.3 | 97.4 |
| 70 | 99.2 | 94.7 | 97.2 |
| The number of features | Low contrast group(Recognition accuracy (%)) | | |
|  | EMD-2DCNN | PPSO | SSD |
| 10 | 99.5 | 98.3 | 97.9 |
| 20 | 99.3 | 98 | 97.5 |
| 30 | 99.2 | 97.4 | 97 |
| 40 | 99.2 | 97.1 | 96.4 |
| 50 | 99.1 | 96.5 | 95.9 |
| 60 | 99 | 95.8 | 95.3 |
| 70 | 98.9 | 94.7 | 95.1 |

**The data in Figure 11**

| Field number | Feature extraction(Running time (ms)) | | |
| --- | --- | --- | --- |
|  | EMD-2DCNN | PPSO | SURF |
| A | 3.2 | 10.8 | 15.1 |
| B | 2.9 | 10.6 | 15.9 |
| C | 4 | 11.4 | 15 |
| D | 4.1 | 11 | 15.8 |
| E | 5.2 | 11.9 | 16.1 |
| F | 3.3 | 11.8 | 14.8 |
| G | 3.1 | 8.7 | 15.2 |
| Field number | Recognition result(Running time (ms)) | | |
|  | EMD-2DCNN | PPSO | SSD |
| A | 4.7 | 13.4 | 25.2 |
| B | 7.2 | 15.1 | 25.6 |
| C | 4.5 | 14.8 | 24.7 |
| D | 4.6 | 13.2 | 23.1 |
| E | 3.3 | 12.8 | 26.8 |
| F | 4.6 | 14.9 | 25.4 |
| G | 6.1 | 13.8 | 24.6 |

**The data in Figure 12**

| Total search times | Single lithium battery failure(The proportion of missearched content (%)) | | | |
| --- | --- | --- | --- | --- |
|  | EMD-2DCNN | PPSO | SURF |  |
| 0 | 0 | 0 | 0 |  |
| 10 | 5.8 | 11.9 | 7.3 |  |
| 20 | 3.9 | 4.1 | 7.1 |  |
| 30 | 4.2 | 6.2 | 11.9 |  |
| 40 | 5.9 | 9.4 | 10.6 |  |
| 50 | 4.1 | 7.8 | 12.4 |  |
| 60 | 4.9 | 8.1 | 12 |  |
| 70 | 5.7 | 7.9 | 15.9 |  |
| 80 | 6 | 9.2 | 14.7 |  |
| 90 | 5.9 | 9.1 | 14.9 |  |
| 100 | 6 | 8.9 | 16 |  |
| Total search times | Lithium battery pack malfunction(The proportion of missearched content (%)) | | |  |
|  | EMD-2DCNN | PPSO | SSD |  |
| 0 | 0 | 0 | 0 |  |
| 10 | 5 | 10 | 4.3 |  |
| 20 | 5.9 | 11.6 | 17.4 |  |
| 30 | 3.8 | 9.9 | 11.4 |  |
| 40 | 6 | 12.1 | 11.3 |  |
| 50 | 4.2 | 8.1 | 8.3 |  |
| 60 | 4.3 | 9.8 | 12.1 |  |
| 70 | 5.7 | 8.6 | 14.2 |  |
| 80 | 5.6 | 10.2 | 11.9 |  |
| 90 | 5.7 | 11.7 | 12.2 |  |
| 100 | 6 | 12.1 | 11.9 |  |

**The data in Figure 13**

| / | Real label A (Proportion (%)) | | |
| --- | --- | --- | --- |
| Prediction label | 1 | 2 | 3 |
| 1 | 100 | 0 | 0 |
| 2 | 0 | 100 | 7 |
| 3 | 0 | 0 | 93 |
|  |  |  |  |
| / | Real label B | | |
| Prediction label | 1 | 2 | 3 |
| 1 | 95 | 0 | 0 |
| 2 | 5 | 100 | 0 |
| 3 | 0 | 0 | 100 |
